# Nesting and Habitat Characteristics of White‐Browed Sparrow‐Weavers (*Plocepasser mahali*, Smith) at Chemeron, Baringo South, Kenya

**DOI:** 10.1002/ece3.73811

**Published:** 2026-06-08

**Authors:** Enock Nyamorambo Nyamira, George Morara Ogendi, Rhoda Nyasuguta Ondieki

**Affiliations:** ^1^ Department of Environmental Science Egerton University Egerton Kenya; ^2^ Dryland Research Training & Ecotourism Centre Chemeron Egerton Kenya

**Keywords:** Arid and semi‐arid lands, Chemeron, habitat degradation, habitat quality, nesting sites, *Plocepasser mahali*

## Abstract

Arid and semi‐arid lands (ASALs) support tropical wooded shrublands and grasslands that sustain a diverse array of flora and fauna, including specialized avian communities. However, as the demand for food, fuel, and other natural resources intensifies, these inherently fragile ecosystems face mounting pressure, resulting in significant habitat degradation and a decline in biodiversity. The objective of this study was to assess and describe the habitat and nesting characteristics of the White‐browed Sparrow‐weavers ( *Plocepasser mahali* ) in the semi‐arid wooded shrublands of Baringo South. This landscape is now heavily modified by anthropogenic activities, including large‐scale charcoal production, harvesting wood fuel, and the spread of agriculture, and is at risk of losing its woody vegetation, which is crucial for birds to nest. To evaluate tree species preference, tree height, diameter at breast height (DBH), and nest height, five (5) ground survey transects were established, radiating from a central point within the study area. The length of these transects varied from 0.82 to 1.79 km, giving a good spatial coverage for evaluating habitat selection and nesting architecture of the White‐browed Sparrow‐weavers. Results indicated that the dominant woody species in order of percent cover were *Acacia tortilis* > *Acacia nilotica* > *Balanites aegyptiaca* > *Acacia elatior.* One‐way analysis of variance (ANOVA) indicated that there was no significant difference in DBH for nesting trees for the *Plocepasser mahali* across the five study transects (*F*_(4,47)_ = 0.63; *p* = 0.65). However, a significant difference in nest height among trees in the five transects was observed (*F*_(4,47)_ = 5.17; *p* = 0.002) suggesting that birds adjust nest placement based on localized vertical structure. Linear regression models showed that diameter at breast height (DBH) had a positive and significant effect on nest numbers (*r* ^2^ = 0.0986; *t* = 2.58; *p* = 0.023). However, no significant relationship was found between basal area (BA) and the number of nests (*r* ^2^ = 0.094; *t* = 0.69; *p* = 0.493). *Acacia tortilis* and *Acacia elatior* were their primary nesting hosts. This preference is driven by the trees' physical structure, with total basal area serving as a key habitat variable that significantly predicts the number of nests across the landscape. This study concludes that habitat characteristics play an important role in the selection of nesting sites by *Plocepasser mahali* . Owing to the overexploitation of *A. tortilis* and *A. elatior* for charcoal and construction, it is important for government agencies to put mechanisms in place to halt their extraction.

## Graphical abstract

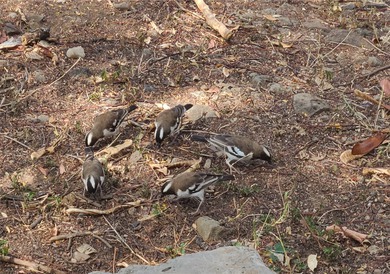

## 1 Introduction

**FIGURE 1:**
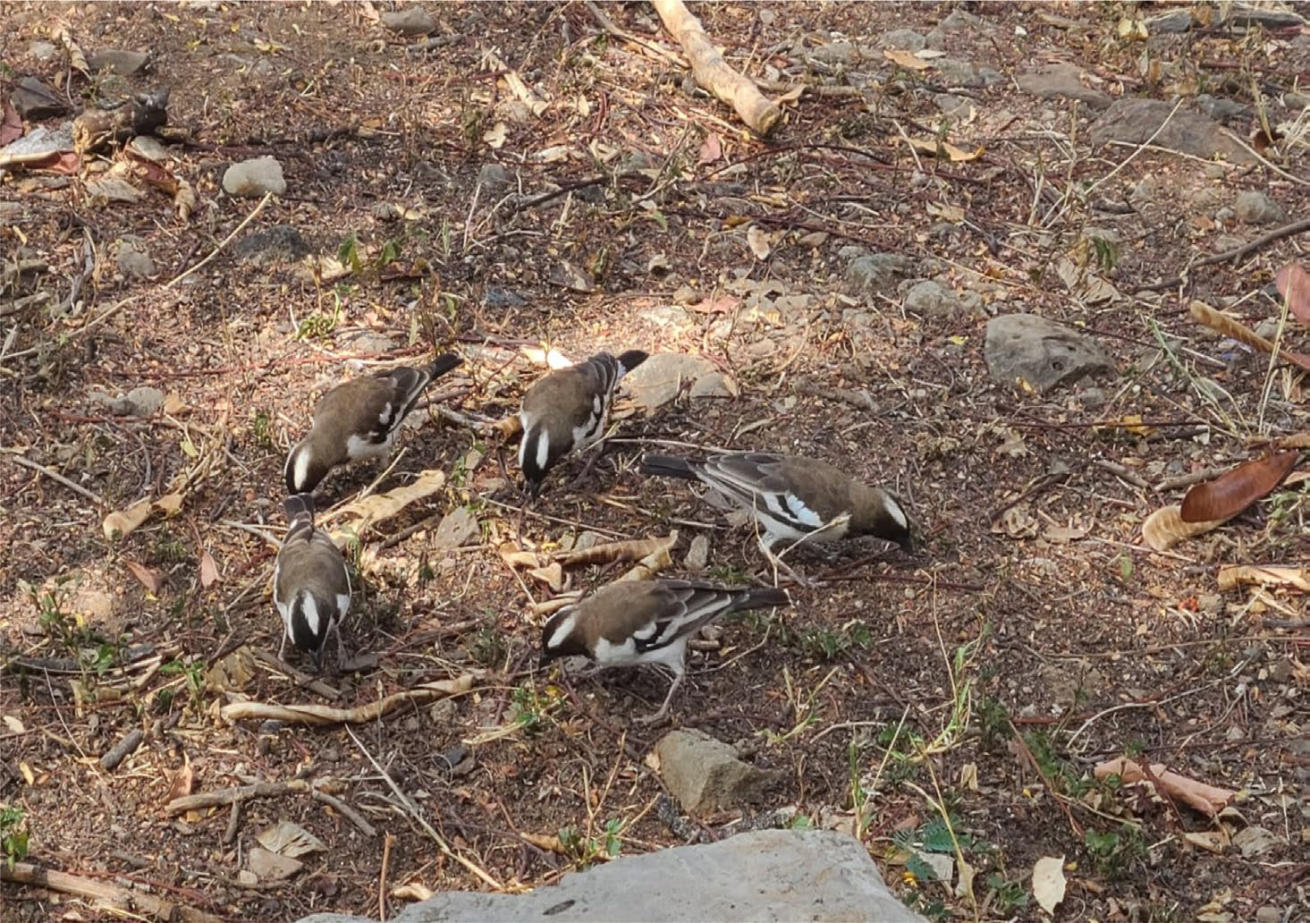
White‐browed Sparrow‐weavers ( *Plocepasser mahali* , Smith) captured at Dryland Research Training and Ecotourism Centre (DRTEC), Chemeron. Photo Credit: Ms. Rhoda Nyasuguta Ondieki.

The White‐browed Sparrow‐weaver ( *Plocepasser mahali* ) is a cooperatively breeding songbird of the genus Plocepasser and family Ploceidae (Figure 1). It is known for making conspicuous, tunnel‐like nests in thorny acacia trees, and is a very important sentinel species in drylands of East Africa because of its noisy persistent alarm calling behavior (Collias and Collias 1978; Harrison et al. 1997). There is however, a significant threat to their populations due to widespread habitat degradation, as these birds are highly dependent on woody vegetation for nesting, shelter and breeding purposes (Bobadoye et al. 2017; Mogaka 2001; United Nations Environment Programme 2009).

This degradation is particularly acute in African arid and semi‐arid lands (ASALs). ASALs are dominated by tropical wooded shrublands and grasslands and encompass more than 40% of the continent, with rich avifaunal communities and hosting over 14 million people in Kenya alone (ASARECA 2011; Ogendi and Ondieki 2020; UNDP 1997). Although these drylands are naturally resilient, a rising demand for food and natural resources has put a lot of stress on the dryland ecosystem. In regions like Baringo South, these anthropogenic pressures manifest as charcoal burning, wood‐fuel harvesting, and agricultural expansion into marginal areas. Ultimately, this structural habitat loss has a negative effect on ecosystem quality, which can be seen as a loss in availability of food, water, and habitat for birds, and consequently a serious effect on avian conservation, reproduction, and survival (Ogendi and Ondieki 2020; Zharikov and Skilleter 2002).

Among the main driving forces of avian community composition are microclimate and habitat structure (Rajpar and Zakaria 2011). A species' population density and nesting locations are often influenced by the type of vegetation, sources of food, and habitat complexity (Norvell et al. 2003). Further, the local climate and habitat features influence how well birds survive, how successfully they breed, when they breed, where they move, and which places they choose to live (Zharikov and Skilleter 2002). Numerous studies have linked the global decline in biodiversity to environmental degradation caused by infrastructure expansion, improper disposal of solid and liquid waste, and urban and agricultural development (Brooks and Thompson 2001; Kettle 2014; Mønkkönen et al. 2014).

Over the last few decades, birds have been recognized as indicators of environmental and climate change, integrating small changes in the ecosystems and thus providing an early warning system to humans about environmental changes. The Afrotropical White‐browed Sparrow‐weavers ( *Plocepasser mahali* Smith), of the family Ploceidae, are birds found in arid areas of north‐eastern and south‐western Africa (BirdLife International 2021; Mackworth‐Praed and Grant 1963; Stevenson and Fanshawe 2002). The species serves as a particularly effective biological indicator due to its high site fidelity and sedentary nature; because colonies are permanent and maintained year‐round, fluctuations in nest density or site abandonment act as a direct proxy for localized habitat degradation and shifts in woody biomass. The White‐browed Sparrow‐weaver is found in dry acacia savanna and wooded shrublands, preferring open, arid habitats characteristic of these habitats, and nesting in small colonies in trees in Kenya (BirdLife International 2021; Collias and Collias 1978; Zimmerman et al. 1999).

The birds dwell in groups of up to eleven, made up of one breeding pair and several non‐reproductive individuals. White‐browed Sparrow‐weavers are social birds whose variations in density have been attributed to differences in food supply and vegetation composition and structure (Norvell et al. 2003; Zharikov and Skilleter 2002). Because their colony stability is highly sensitive to the structural health of the *Acacia* overstory, they provide measurable insights into the broader ecological impacts of anthropogenic pressure. The average size of a colony of *P. mahali* is 16 to 20 individual grass structures that are maintained year round (Stevenson and Fanshawe 2002; Zimmerman et al. 1999). These structures must be distinguished from each other because most are not active nests but roosting places. These colonies have two functional structures: breeding nests with one entrance and roosting nests with two entrances for quick escape (Earle 1983). In this study, the total number of these structures is referred to as “total nests” or “communal structures”, and their specific functions are distinguished where possible to avoid overestimating breeding activity. The ratio and location of these particular structures are key aspects of habitat quality and are important to understand in the context of the environment. In terms of conservation status, it is considered Least Concern (LC) owing to its large population and wide distribution. It is however worth recognizing that while they are not a threatened species, their habitats are increasingly being degraded particularly in developing countries where human encroachment and settlement in fragile arid environments (BirdLife International 2021; International Union for Conservation of Nature 2024).

Like many other birds in the ASALs, *P. mahali* plays multiple roles including seed dispersal, recreation, education, and an indicator of environmental and climate change (Altwegg et al. 2008; Ogendi and Ondieki 2020). Despite their role in environmental conservation, this species like the rest is facing intense pressure stemming from human activities in the study area (this include, charcoal burning, wood fuel extraction, human settlements, and agricultural expansion). Currently, there is limited knowledge on its nesting and habitat characteristics in the presence of increasing anthropogenic pressure in the study area that lies within Baringo South, Kenya (United Nations Environment Programme 2009; Western et al. 2015; Government of Kenya 2013). Although the region plays an important role in conserving both global and regional biodiversity, the use of valuable large trees like *Acacia tortilis, Acacia seyal, Acacia elatior, Acacia nilotica, Terminalia brownii*, and *Boscia angustifolia* for charcoal production, building poles, and fencing is gradually lowering tree cover and available food for birds, putting strain on their survival. Chemeron is located approximately 20 km from Lake Bogoria, a UNESCO World Heritage Site, Important Bird Area, and wetland of international importance (Ramsar Site) (Ogendi et al. 2025). Thus anthropogenic activities in the study area are likely to have negative effects on avian assemblages in and around Lake Bogoria National Reserve (LBNR). There is limited information on the nesting ecology of *P. mahali* in Kenya which could otherwise assist in its conservation and that of its habitat.

The White‐browed Sparrow‐weavers ( *Plocepasser mahali* ) are an important model species for dryland health monitoring in Afrotropical savannas and are currently classified as “Least Concern” on the IUCN Red List. These birds are very dependent upon the structure and density of the vegetation required for communal nesting colonies, making them a biological “early warning system”. By examining their nesting habits, we can gain insights into the cascading effects they have when their key tree species, like Acacia, are lost. These observations can be used to improve our understanding of the broader ecological consequences of habitat loss, particularly on avian biodiversity in fragile ASAL habitats (BirdLife International 2023; IPBES 2019; Sekercioglu et al. 2016). This study therefore aimed to assess and describe the habitat and nesting characteristics of *P. mahali* in the semi‐arid wooded shrublands of Baringo South. In particular, habitat characterization consisted of measuring the woody species composition, tree density, and herbaceous ground cover dominance using the Importance Value Index (IVI). Nesting characteristics were assessed by recording nest tree species and height, Diameter at Breast Height (DBH), nest density per nest tree, and architectural differences between breeding and roosting nests. This objective was addressed by exploring three questions: (i) What are the habitat characteristics of the White‐browed Sparrow‐weavers ( *P. mahali* ) within the study area? (ii) What are the nesting characteristics of the White‐browed Sparrow‐weavers? (iii) Are there salient relationships between selected habitat characteristics and the nesting of White‐browed Sparrow‐weavers?

## 2 Methodology

### 2.1 Description of Study Area

**FIGURE 2:**
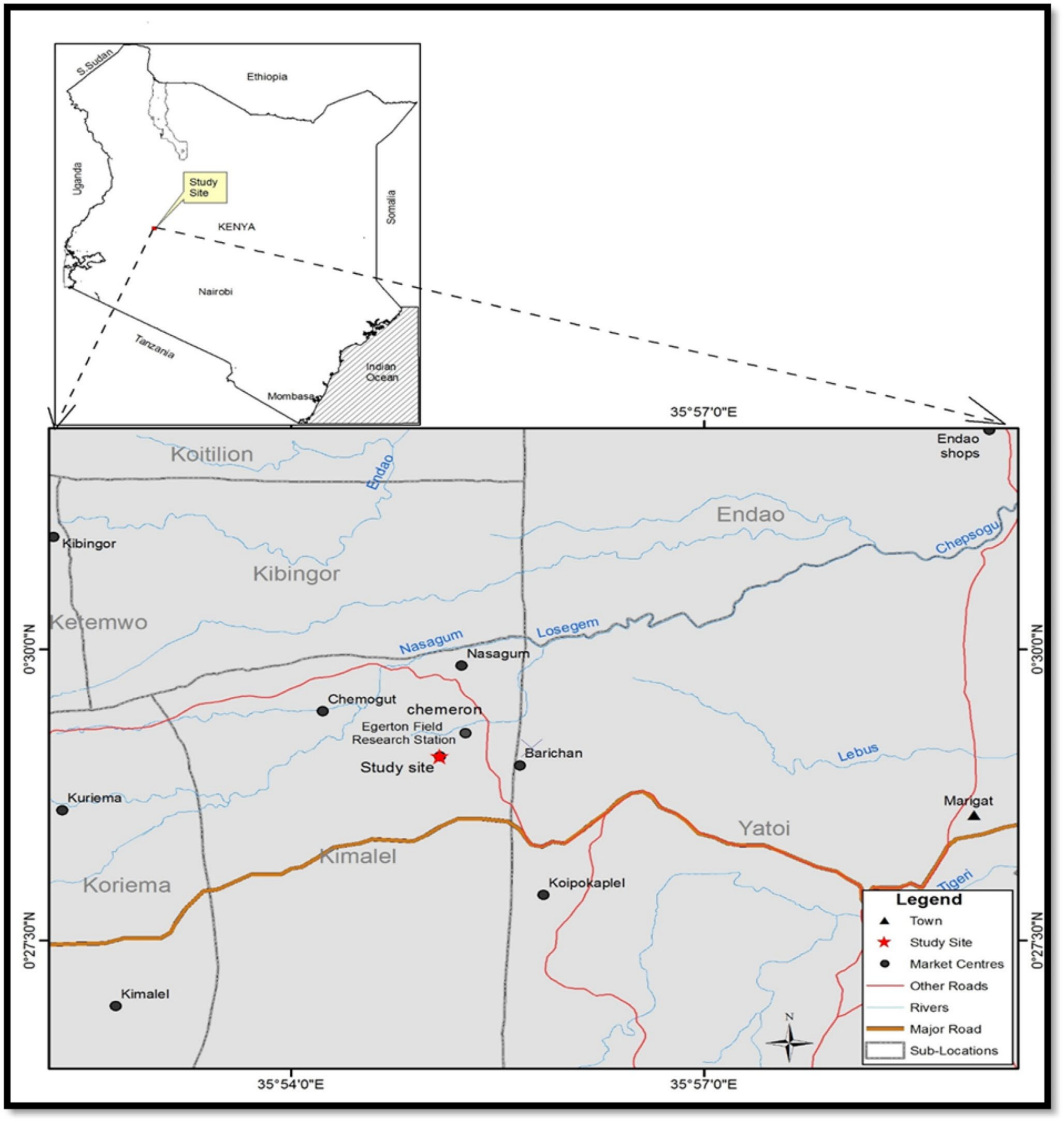
Map of Baringo South Sub‐County (Map of Kenya inset) showing the study transects (Chemeron).

This study on the White‐browed Sparrow‐weavers was carried out at the Chemeron Dryland Research, Training, and Ecotourism Centre (Figure 2), that is approximately 300 km west of Nairobi City, and approximately 30 km from Kabarnet town in Baringo County. The study area is situated in Agro‐Ecological Zone V, which receives an average annual rainfall of 635 mm and lies at an altitude of around 1200 m above sea level. The soils are described as reddish‐brown sandy loam with numerous rocky outcrops that support rangeland vegetation and sustain livelihoods such as beekeeping and livestock production (Ogendi et al. 2025). The primary source of income for the local inhabitants is agro‐pastoralism, which includes rearing goats, cattle, and apiculture, due to the area's underdeveloped soils. A combination of stony, shallow sandy soils, frequent droughts, and little rainfall make it difficult to grow commercial crops (Ogendi and Ondieki 2020). Short‐season and drought‐tolerant crops such as finger millet, pearl millet, pigeon peas, vegetables, and fruits like mangoes, pawpaws, and lemons can be grown in the area. However, the area has no permanent rivers or water sources. The main sources of water are boreholes and water pans, the latter proving to be an important factor that influences the distribution of wildlife and livestock within the study area. The study location is situated between Lake Bogoria and Lake Baringo, two significant bodies of water, approximately 30 km and 25 km away, respectively.

Vegetation in the study area can be classified as wooded savannah shrublands mainly dominated by acacia species (Ogendi and Ondieki 2020). Among the acacia species are *Acacia reficiens*, *Acacia brevispica*, *Acacia Senegal* , *Acacia mellifera* , *Acacia tortilis* , *Acacia elatior*. The last two species constitute the large and tall woody species that are preferred for woodfuel and construction which also constitute the preferred nesting sites for local birds. The area is also interspersed with other trees and shrubs that are important as livestock feed/browse and herbal medicine to the local community (Ogendi et al. 2025). They include *Boscia anguistifolia, Boscia coriacea, Tecla trichocarpa, Balanites aegyptiaca, Grewia bicolor, Terminalia brownii, Maerua angolensis, Maerua kirkii, Acalypha fruticosa* and *Berchemia discolor*. The dominant grass species in the study area are *Aristida keniensis, Chloris roxburghiana* and *Eragrostis superba*. Buffel grass ( *Cenchrus ciliaris* ) has been introduced in the expansive 1100‐acre Egerton University land to enhance fodder for livestock (Ogendi and Ondieki 2020). Five transects were chosen for this study, comprising wooded shrublands and wooded grasslands. The GPS coordinates, vegetation composition and characteristics for the study transects are detailed in Table 1. This study was carried out along five radiating transects in Chemeron, Baringo South. These data are essential for understanding the distribution of *P. mahali* colonies along the various ecological zones and anthropogenic gradients sampled.

**TABLE 1:** GPS coordinates, dominant habitat types, transect lengths, and altitude ranges for the five study transects in Dryland Research Training and Ecotourism Centre (DRTEC), Chemeron, Baringo South.

Transect name/length/M.s.l	GPS coordinates		Dominant plant species
	Start	End	
Transect 1 0.82 km^a^ 1213–1221 m^b^	825212.46 E 54145.84 N	825173.59 E 53920.32 N	*Acacia senegal*, *Acacia nilotica*, *Zizyphus mucronata*, *Cordia sinensis*, *Cenchrus ciliaris*, *Aristida keniensis*, *Leuceana leucocephala*, *Delonix regia*, *Azadiracta indica*, and *Acacia Senegal*, *Balanites aegyptiaca*, *Brachiaria ruziziensis*, *Cynodon dactylon* (Adjacent office buildings/hostels/Tree Nursery/rehabilitated land)
Transect 2 0.85 km^a^ 1212–1209 m^b^	825210.39 E 53852.23 N	825347.49 E 53959.22 N	*Acacia tortilis*, *Acacia nilotica*, *Acacia mellifera*, *Acacia brevispica*, *Aristida keniensis*, *Acacia senegal*, *Berchemia discolor*, *Acalypha fruticosa* (Within Chemeron DRTEC Nature Park)
Transect 3 1.49 km^a^ 1211–1210 m^b^	825329.38 E 54024.00 N	825240.19 E 54375.19 N	*Acacia senegal*, *Boscia angustifolia*, *Zizyphus mucronata*, *Grewia bicolor*, *Cenchrus ciliaris*, *Aristida keniensis*, *Acacia elatior*, *Acacia nilotica*, *Azadiracta indica*, and *Acacia Senegal*, *Acalypha fruticosa*, *Brachiaria ruziziensis*, *Cynodon dactylon* (Adjacent Main Gate to Chemeron DRTEC)
Transect 4 1.79 km^a^ 1208–1225 m^b^	825190.21 E 54347.25 N	825040.87 E 54122.55 N	*Acacia tortilis*, *Acacia elatior*, *Maerua angolensis*, *Zizyphus mucronata*, *Cordia sinensis*, *Cenchrus ciliaris*, *Aristida keniensis*, *Azadiracta indica*, and *Acacia Senegal*, *Balanites aegyptiaca* *Acacia senegal‐ Balanites aegyptica* (Adjacent Staff Quarters and Apiary)
Transect 5 0.95 km 1207–1193 m^b^	825143.54 E 54419.78 N	824935.82 E 54650.36 N	*Acacia elatior*, *Balanites aegyptica*, *Zizyphus mucronata*, *Cordia sinensis*, *Tamarindus indica*, *Acacia senegal*, *Boscia angustifolia*, *Grewia bicolor* (Towards Chemeron River)

*Note:* Superscripts a and b indicate transect length and altitude range, respectively.

### 2.2 Research and Sampling Design

Ground surveys of the White‐browed Sparrow‐weavers were done along five (5) transect lines ranging from 0.82 to 1.79 km in length, radiating from a central point within the study area. These lines were not randomly placed; they were systematically and purposefully oriented to provide an even and representative sample of the landscape and to include a clear gradient of anthropogenic disturbance. The radiating design was deliberately selected to sample a gradient of anthropogenic disturbance from the high activity central research area to the relatively undisturbed nature park boundaries. All nesting data were obtained along these same five radiating transects to provide spatial uniformity. Observations were made during times of peak avian activity, as described by Bennun et al. (2002): 06:30 to 09:30 and 16:00 to 18:00. Fieldwork was conducted from December 2020 to May 2021.

Transects were traversed at a steady pace, and all relevant data were recorded in field notebooks. The communal grass nests of *P. mahali* were conspicuous, but a systematic approach was needed to identify all nests, particularly those hidden by thick vegetation. To ensure a complete census, two observers with binoculars (8 × 42) scanned the canopy of each woody individual within a 25 m buffer on either side of the center‐line of the transect. A handheld Garmin GPS (Map 64 s) was used to record the general habitat type, current weather conditions, evidence of human activity (e.g., livestock presence) and start/end GPS coordinates at the beginning of each survey. Two common ecological sampling techniques were used to quantify the habitat composition along these transects. The Point‐Centered Quarter (PCQ) method was used to measure density and frequency of woody species (trees and shrubs) at 100 m intervals. In each quadrant, the species of the nearest woody individual was identified, and its distance from the sampling point was measured and recorded. At the same time, the dominance of the herbaceous and grass layer was determined by a 0.5 m × 0.5 m, Daubenmire frame set at 50 m intervals. The percentage canopy cover of each grass species was visually estimated in each frame to calculate the Importance Value Index (IVI) for the site.

The nesting and habitat characteristics of White‐browed Sparrow‐weavers were systematically recorded. In this study, “nest density” was defined as the number of individual communal nest structures per host tree. We identified the species and Diameter at Breast Height (DBH) at 1.3 m with a metric diameter tape for each nesting tree (*n* = 52). Tree height and nest height were measured with a Suunto clinometer from a fixed distance of 15 m, to the nearest 0.1 m. A comparative method was used to assess host tree selection by measuring the height of the nearest non‐nesting tree (within 10 m) with the same equipment and method. The compass direction (azimuth) of the main entrance hole was used to determine nest orientation and was recorded with a hand‐held magnetic compass. This data was collected to evaluate the birds' strategies for microclimate regulation and predator avoidance. Orientation is a very important adaptive feature; in arid areas, birds may orient entrance holes to prevent the internal chamber from being buffeted by wind, or to control thermal exposure from direct solar radiation (Ferguson and Siegfried 1989). Breeding nests (single entrance) were distinguished from roosting nests (two entrances) following Collias and Collias (1978) and a total of 424 nests were recorded.

### 2.3 Data Collection Protocols

Nesting and habitat features of White‐browed Sparrow‐weavers were systematically observed and recorded. A multi‐step measurement protocol similar to Ferguson and Siegfried (1989) was used to ensure a standardized assessment. The Diameter at Breast Height (DBH) was measured at 1.3 m above the ground on each identified nesting tree with a standard metric diameter tape. Tree height and the height of each nest were measured with a Suunto clinometer from a fixed distance of 15 m to ensure vertical accuracy. The height of the four nearest neighboring trees (non‐nesting) within a 10 m radius was measured using the same clinometric method to evaluate the selection of host trees in relation to the surrounding vegetation. Nest orientation was calculated by setting a compass to the direction of the center‐line of the entrance hole from the point directly below the nest and reading the direction to the nearest degree (0°–360°). This data was gathered to assess the birds' approach to microclimate regulation; in dry habitats, the orientation of the entrance is an important adaptive feature to protect the internal chamber from the wind or to control thermal exposure from direct solar radiation (Ferguson and Siegfried 1989).

In addition to the classification by Collias and Collias (1978), we separated breeding nests (single entrance) from roosting structures (two entrances) to avoid combining the majority of builds, which serve as year‐round roosts, with active breeding nests. The distance from each nesting tree to the closest permanent water source was also measured from each tree using a handheld Garmin GPS (Map 64 s) which has a ±3 m accuracy. A total of 424 nests were documented at the end of the survey, in 52 trees, with placement, orientation, and habitat characteristics associated with each nest. Tree Basal Area (BA) was determined using the formula for the area of a circle ($A=\pi r^{2}$ 2 where *r* = radius and π = 3.142) and the calculation for radius (*r* = diameter/2 = DBH/2). DBH is the diameter at breast height. Transects were employed to provide systematic coverage, but the study area was considered as a single landscape unit and five subsamples were used to compensate for the spatial overlap at the central radiation point. This method allowed for a spatially stratified sampling, but the results were a unified analysis of the local population's response to habitat characteristics.

### 2.4 Data Analysis

All statistical analyses were conducted using Minitab (2000) and R software, with a significance threshold set at *α* = 0.05 (Sokal and Rohlf 1995). Exploratory data analysis was conducted before hypothesis testing to evaluate the distribution of the data and to check the assumptions for parametric tests. The Kolmogorov–Smirnov (*p* > 0.15) and Levene's tests (*p* > 0.05) were used to test the normality of the data and model residuals, respectively, and the data were found to be suitable for parametric modeling. Diagnostic plots such as Q–Q plots and Residuals versus Fitted plots were also examined visually for normality of residuals and homoscedasticity. If assumptions for parametric analysis were not met, data were log transformed or non‐parametric alternatives were considered. A one‐way Analysis of Variance (ANOVA) was used to determine whether significant differences existed in mean nest height, tree DBH, and basal area across the five (5) transects. Survey lines were not considered as independent sites because of potential spatial dependence among the radiating transects. To determine the relative importance of total basal area, DBH, and distance to water, multiple linear regression models were also used to determine the major factors influencing nest density (number of nests per tree) in the selection of nesting sites. The coefficient of determination (*r* ^2^) and standardized regression coefficients were used to assess the predictive power of each habitat variable. All results are presented with full statistical notation, including *F‐*statistics, degrees of freedom (df) and exact *p*‐values, for transparency and reproducibility.

## 3 Results

### 3.1 Habitat Characterization for Nesting Sites

The survey area covered an elevation range from 1208 to 1225 m above sea level (Table 1). The study site is quantitatively a mosaic of Acacia‐wooded shrublands and Acacia‐wooded grasslands with a clear vertical and horizontal structural hierarchy. The term “dominant” is used here as a quantitative ecological descriptor. It is defined as number of individuals (*n*) in the overstory and as percent cover in the shrub and grass layers. A total of 208 individuals were recorded using the Point‐Centered Quarter (PCQ) method of analyzing the woody overstory. These were dominated by a suite of xeric adapted species in the following order: *Acacia tortilis* (*n* = 84), *Acacia nilotica* (*n* = 52), *Balanites aegyptiaca* (*n* = 41), *Acacia elatior* (*n* = 31). *A. nilotica* and *B. aegyptiaca* were the second and third most abundant species in the landscape, but not used for nesting. *P. mahali* , however, strongly favored *A. tortilis* and *A. elatior* , the two species that together supported most of the colonies, although *A. elatior* was the least common of the four dominant species. This is evidence that tree selection is not just a matter of local availability but is instead determined by a structural suitability. A similar pattern was observed in the mid‐story shrub layer, where *Acacia senegal* (32% relative cover), *Acacia brevispica* (24% relative cover) and *Acalypha fruticosa* (15% relative cover) were the most abundant species.

One of the most important results of this study is the extreme taxonomic and functional specificity of nest‐building resource selection in relation to biomass available. The herbaceous layer was rich in potential materials, but when cover estimates were obtained by the Daubenmire method, four species were found to be dominant: *Aristida keniensis* (42% mean cover); *Cenchrus ciliaris* (28% mean cover); *Brachiaria ruziziensis* (15% mean cover); and *Cynodon dactylon* (10% mean cover). In spite of this diversity, *P. mahali* showed a near exclusive preference for one grass species. Over 95% of the nests were constructed using *A. keniensis*. Notably, *Cenchrus ciliaris* was entirely excluded from nest construction, despite being the second most abundant grass species recorded in the study area. This non‐random choice implies that *P. mahali* is assessing the mechanical properties of the nesting material in question. The preference for *A. keniensis* is probably due to the better flexibility, tensile strength and “hooking” properties of its stems. They are all functional in the construction of the species' complex, tunnel‐shaped nests and in keeping them securely in the thorny canopy of host trees. The high density of these particular nesting materials and host resources emphasizes the high carrying capacity of the study area and the importance of the area as a critical habitat for *P. mahali* in the increasingly fragmented Baringo landscape.

### 3.2 Tree Selection Preferences

A comparison of nesting trees with the nearest surrounding trees found a strong preference for taller canopy structures. Nesting trees were significantly taller (6.66 ± 0.26 m) than adjacent non‐nesting trees (4.90 ± 0.17 m) (Paired *t*‐test: *t* = 8.16, df = 51, *p* < 0.001; Figure 13). This indicates that height is a major selection criterion for *P. mahali* colony establishment. In addition, the preferred host species, mostly *A. tortilis* and *A. elatior* , were an architectural class with dense umbrella‐shaped canopies and strong stipular thorns which were significantly diminished or missing in adjacent non‐nesting woody species. This indicates that species with the greatest vertical clearance and best physical defense against climbing predators were selected.

### 3.3 Nesting Site Characteristics

**FIGURE 3:**
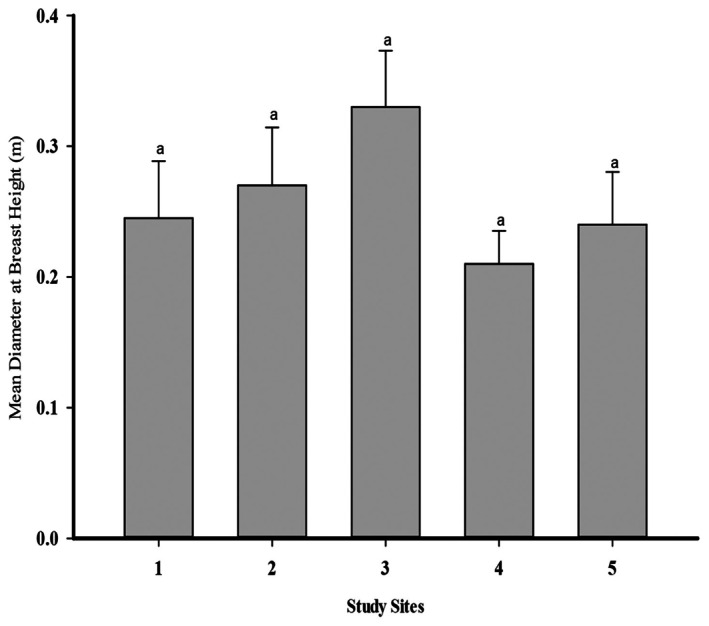
Mean Diameter at Breast Height for nesting trees in the five study transects at Chemeron, South Baringo, Kenya. Transects (Means) with the same letter are not significantly different from one another (*p* > 0.05).

**FIGURE 4:**
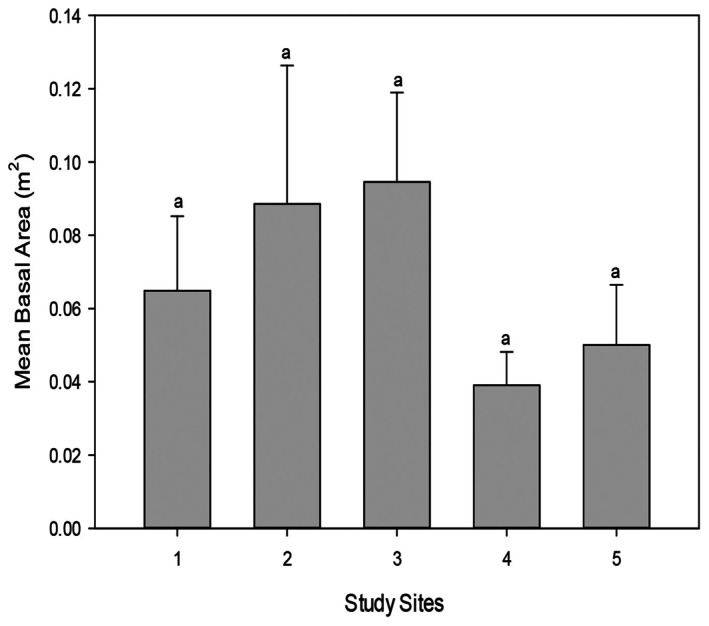
Mean nesting tree Basal Area at the five study transects at Chemeron, South Baringo, Kenya. Transects (Means) with the same letter are not significantly different from one another (*p* > 0.05).

**FIGURE 5:**
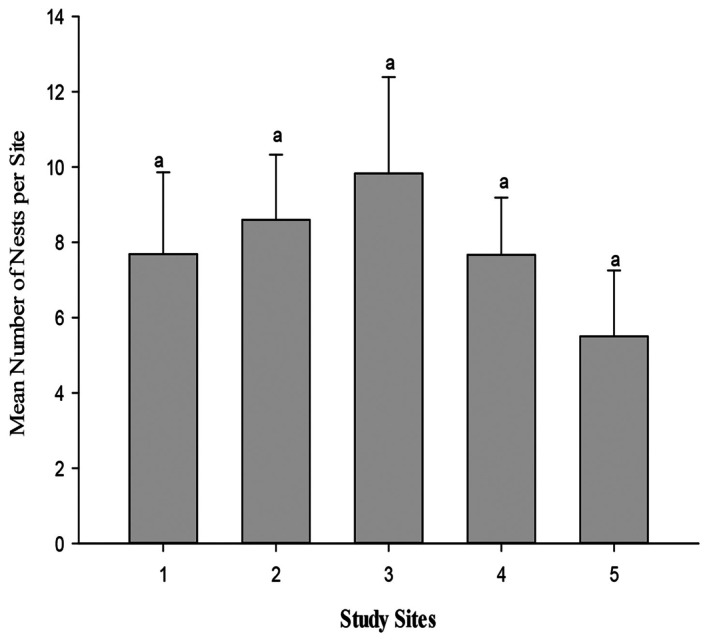
Mean number of nests per tree in each of the study transects at Chemeron, South Baringo, Kenya. Transects (Means) with the same letter are not significantly different from one another (*p* > 0.05).

**FIGURE 6:**
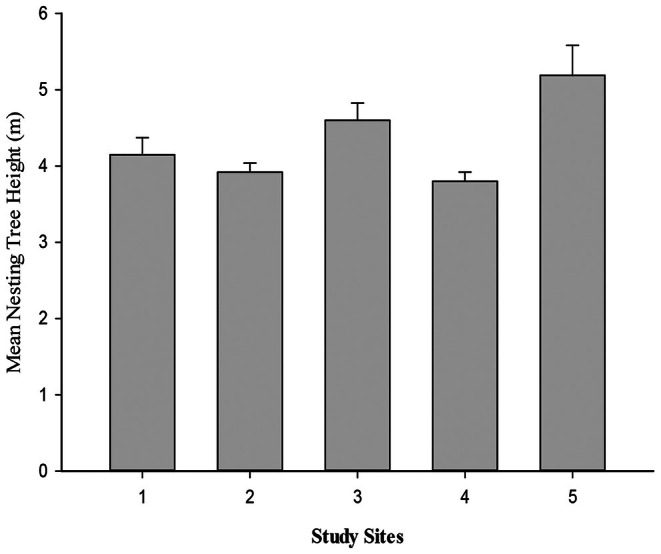
Mean Nest Height at the five study transects at Chemeron, South Baringo, Kenya. Transects (Means) with the same letter are not significantly different from one another (*p* > 0.05).

**FIGURE 7:**
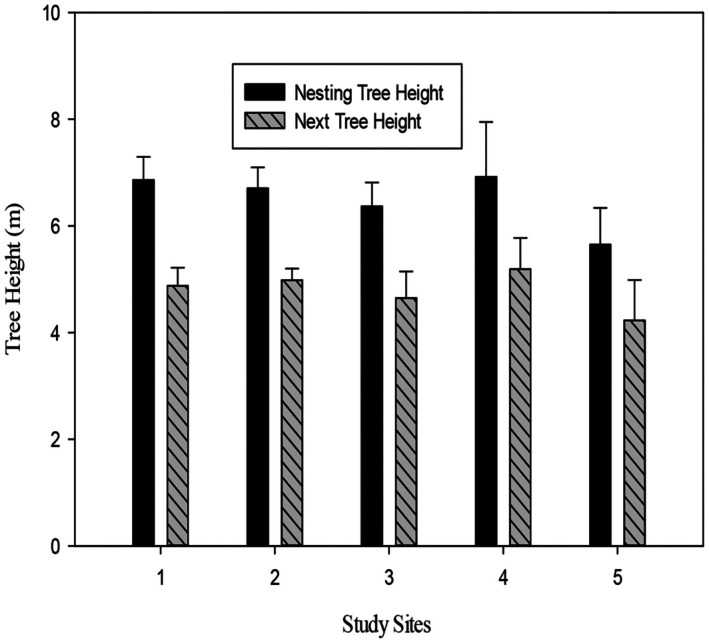
Nesting tree height variation for *P. mahali* in Baringo South, Kenya.

**FIGURE 8:**
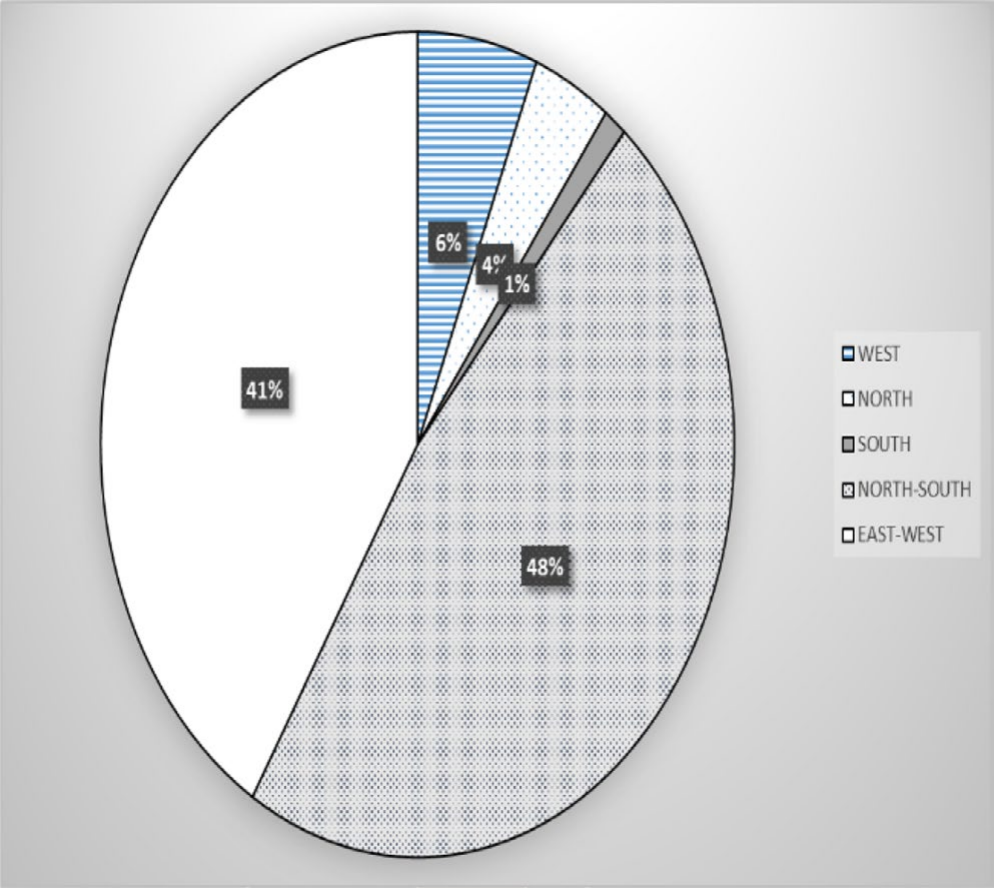
Nest orientation for the *P. mahali* in the five transects in Baringo South, Kenya.

The size of host trees was fairly consistent throughout the study area. Mean Diameter at Breast Height (DBH) ranged from 0.21 ± 0.025 m (mean ± SE) to 0.33 ± 0.043 (mean ± SE) (Figure 3), whereas the mean basal area ranged from 47.31 ± 6.647 to 77.71 ± 13.799 (Figure 4). The White‐browed Sparrow‐weavers ( *Plocepasser mahali* ) did not show any significant difference in the DBH of trees they nested on among the five study transects (*F*_(4,47)_ = 0.63; *p* = 0.65; Figure 3). This spatial homogeneity of host tree size was confirmed by Tukey's pairwise comparisons at *p* = 0.05. Similarly, no significant differences were observed in the basal area of nesting trees (*F*_(4,47)_ = 0.92; *p* = 0.46; Figure 4) or the mean number of nests per tree (*F*_(4,47)_ = 0.28; *p* = 0.89; Figure 5). Nest placement height differed significantly between the five transects (*F*_(4,47)_ = 5.17; *p* = 0.002; Figure 6), but not tree dimensions. A Tukey's pairwise comparison test revealed that nests in transects 3 and 5 were significantly higher than nests in transects 1, 2, and 4. Interestingly, this variation in nest height was not a result of differences in tree availability, as overall nesting tree height did not differ significantly among transects (*F*_(4,47)_ = 0.39; *p* = 0.82). Furthermore, there were no significant differences between the distance of the nesting trees and the closest water source (*F*_(4,47)_ = 1.12; *p* = 0.36; Figure 7). A significant structural preference was noted: host trees were always taller than surrounding non‐nesting woody plants, thus providing greater visibility and space for the birds to fly from tree to tree (Figure 7). Colony composition: Roosting nests (two entrances) accounted for 89% of the nests, while breeding nests (one entrance) accounted for 11% (Figure 8). These structures were highly non‐randomly oriented, as shown in the radar chart (Figure 14). General cardinal directions were noted, but there was a significant concentration of nests on the South‐West (SW) aspect of host trees (*p* < 0.001). This particular orientation is an important microhabitat preference for the leeward side of the canopy as compared to the prevailing North‐Easterly winds of the Baringo County landscape. Additionally, 85% of nests were located in the upper two‐thirds of the canopy, utilizing the dense foliage of *A. tortilis* and *A. elatior* for overhead shading.

### 3.4 Relationships Among Various Nesting Characteristics and Number of Nests

**FIGURE 9:**
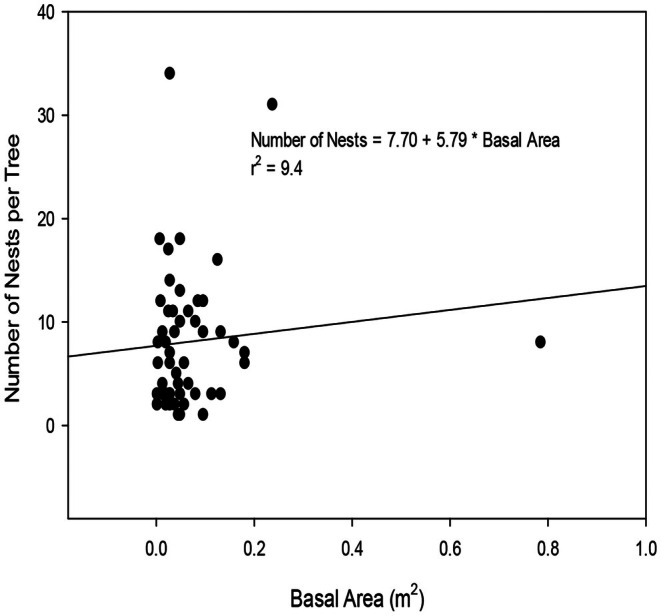
Relationship between Basal Area and Number of Nests of *P. mahali* on nesting trees in Baringo South, Kenya.

A series of regression analyses were conducted to establish the influence of various dendrometric and environmental variables on the total number of nests per tree. To ensure a full appreciation of the model's predictive power, we report the regression equations, coefficients of determination (*r* ^2^), and *t*‐statistics for each parameter. The relationship between total basal area and nest abundance showed no significant correlation (the model indicated that basal area positively and significantly influenced the number of nests; Regression Equation: Number of nests = 7.70 + 5.79 × Basal Area; *r* ^2^ = 0.094; *t* = 0.69; *p* = 0.493; Figure 9). While the model suggested that basal area accounted for approximately 9.4% of the observed variation, the high *p*‐value indicates that basal area is not a reliable predictor of colony size within this landscape.

**FIGURE 10:**
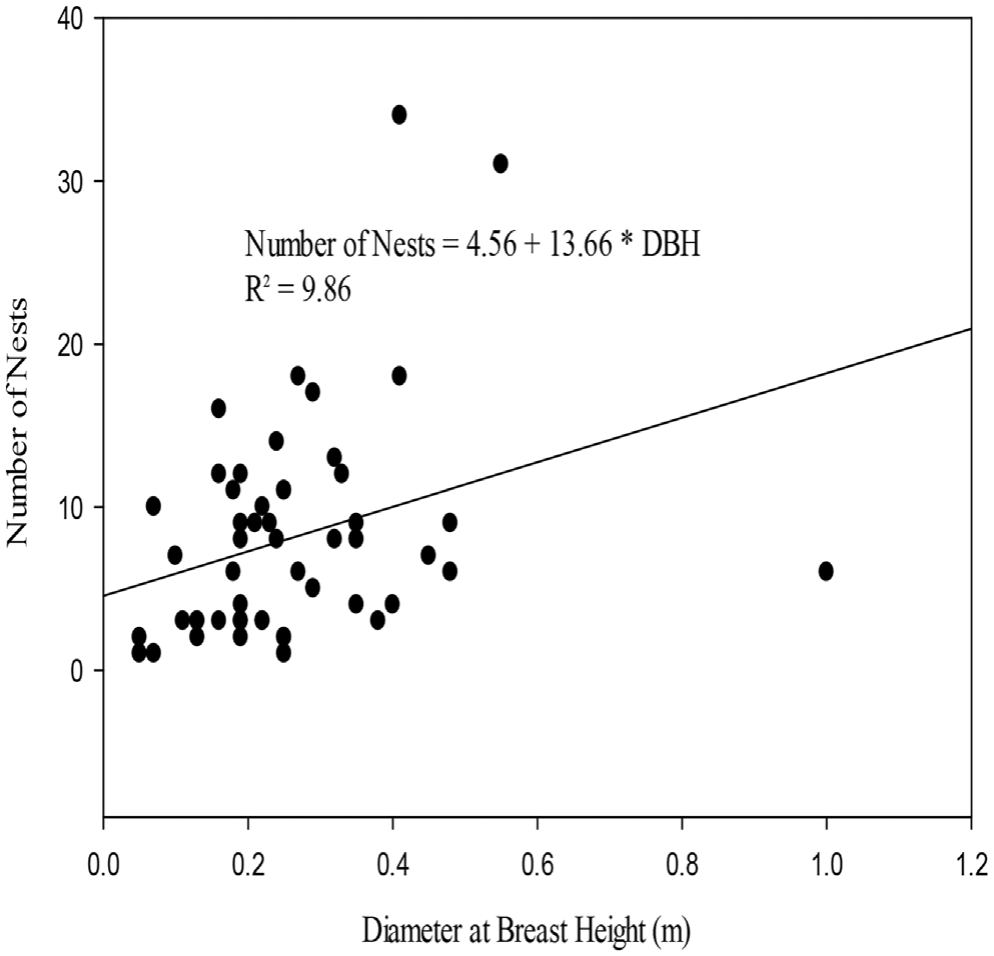
Relationship between DBH and Number of Nests of *P. mahali* on nesting trees in Baringo South, Kenya.

In contrast, Diameter at Breast Height (DBH) was identified as a significant positive predictor of the number of nests per tree (Regression model: Number of nests = 4. 56 + 13.66 × DBH; *r* ^2^ = 0.0986; *t* = 2.58; *p* = 0.023; Figure 10). Therefore, the regression model demonstrated that DBH can account for approximately 10% of the variation in the number of nests for the *Plocepasser mahali* in the study area, suggesting that as tree girth increases, the physical capacity of the tree to support larger communal structures also increases.

**FIGURE 11:**
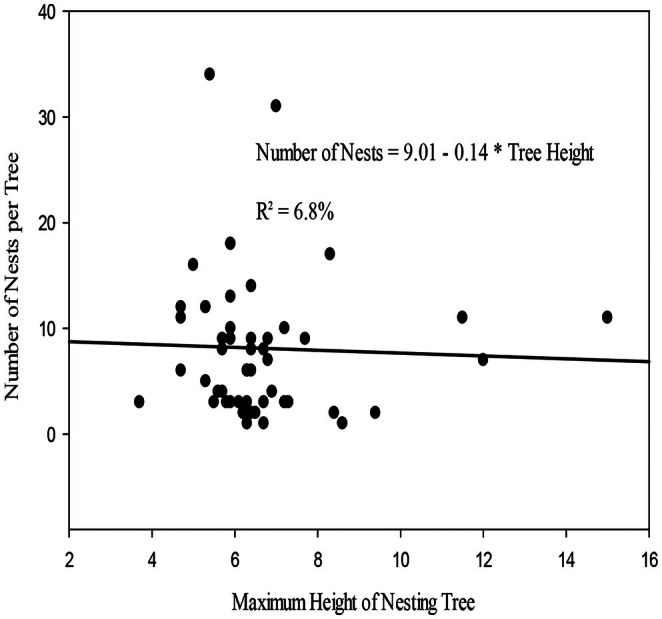
Relationship between maximum height of nesting tree and number of nests of *P. mahali* on nesting trees in Baringo South, Kenya.

A regression analysis was conducted to establish the influence of tree height of nesting trees on several *Plocepasser mahali* nests on a tree. Conversely, tree height did not significantly influence the number of nests (Regression model: Number of nests = 9.012–0.14 × Tree Height; *r* ^2^ = 0.068; *p* = 0.791; Figure 11). The negative coefficient and non‐significant *p*‐value suggest that once a minimum height threshold is reached for nesting, further increases in tree height do not result in higher nest counts.

**FIGURE 12:**
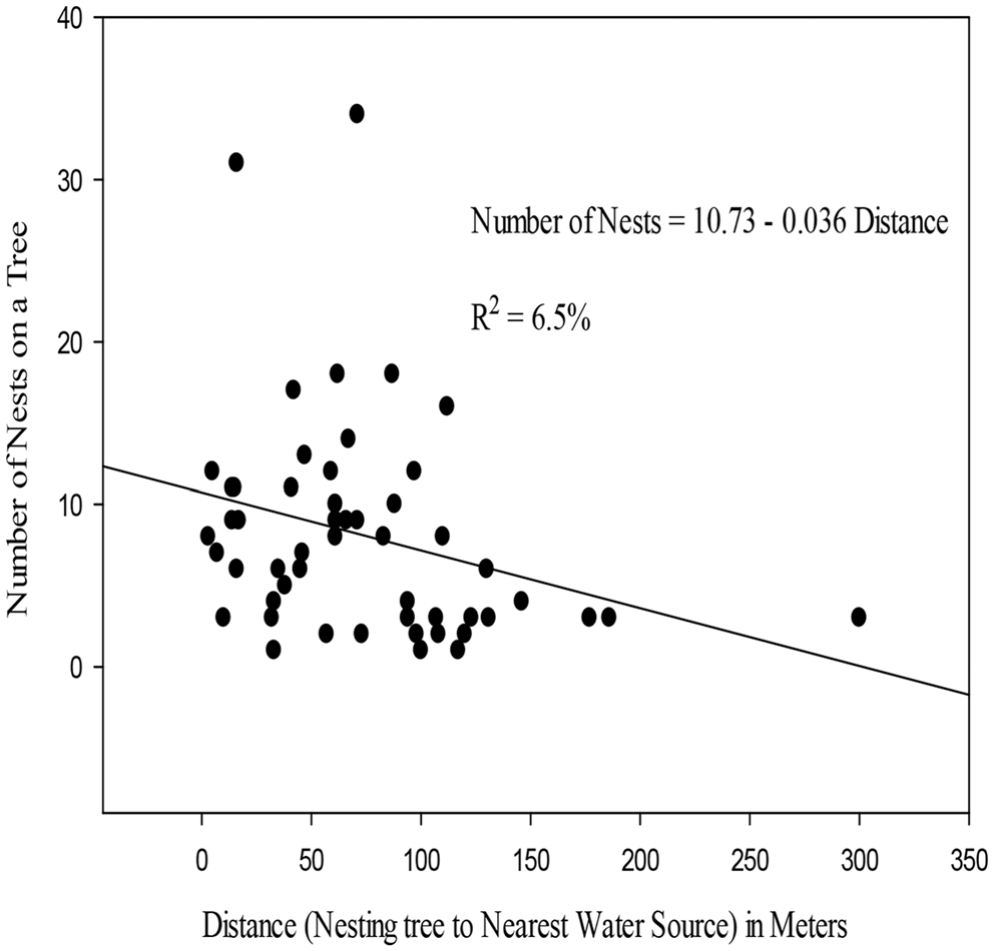
Relationship between nearest Source of Water and Number of Nests of *P. mahali* on nesting trees in Baringo South, Kenya.

Finally, another regression analysis was conducted to establish the influence of distance to the nearest water source on the number of nests on a tree in the study area. The model indicated that distance to the water source could be used to predict the number of nests on a nesting tree (Regression model: Number of nests = 10.73 + 0.06 × Distance; *r* ^2^ = 0.065; *p* = 0.032; Figure 12). This suggests that while proximity to water is a known factor in avian habitat selection, the spatial distribution of *P. mahali* colonies in Baringo South is significantly influenced by the distance to permanent water points, accounting for approximately 7% of the nesting variance for the *Plocepasser mahali* in the study area. The inclusion of these *p*‐values and *r* ^2^ metrics provides the detail necessary for the results to be fully evaluated and compared with other studies in semi‐arid ecosystems.

## 4 Discussion

### 4.1 Habitat Modification and Anthropogenic Influence

Spatial distribution of nesting sites showed preference in the present study for sites with visible human disturbance. In particular, the rangeland enclosures and habitat manipulations near the research center seem to have established optimum conditions for *P. mahali* colonies. The nesting density in these managed rangelands is likely to be partly due to the year‐round availability of herbaceous and grass species, which contributes to a high foraging visibility in the landscape. The diverse diet of these birds, including termites, weevils, beetles, ants, caterpillars, and small moths, makes the modified habitat suitable for White‐browed Sparrow‐weavers to nest and breed (Ferguson and Siegfried 1989; Stevenson and Fanshawe 2002; Zimmerman et al. 1999).

Birds of this species have demonstrated impressive adaptability, allowing them to thrive in the open rangelands of arid Africa. Their diet, rich in termites, has been a key factor in their success (Bouillon 1970). The structure and type of vegetation are critical in nest site selection for most bird species (Jayapal et al. 2009; Maclean 1993; Murray and Best 2014). Owing to this requirement in siting of nests, the survival of most bird species is at risk given the increasing human population coupled with intense pressure for land for human settlements, demand for biomass fuels, and expansion of agriculture. Therefore, alterations in the composition and structure of vegetation that constitute nesting habitats may significantly affect the survival of birds. This threat is real and present in the study area and its environs owing to increased extraction and utilization of *Acacia elatior* and *Acacia tortilis* by the local residents for charcoal and building materials. Both species are favored for charcoal production because they have the highest calorific values of 4400 and 5394 Kcal for *A. tortilis* and *A. elatior* respectively (Orwa et al. 2009). In such landscapes, the most structurally suitable trees for birds to nest in are most profitable for human energy use, resulting in a “resource bottleneck” in degraded landscapes.

### 4.2 Selection of Host Tree Species and Structural Architecture

**FIGURE 13:**
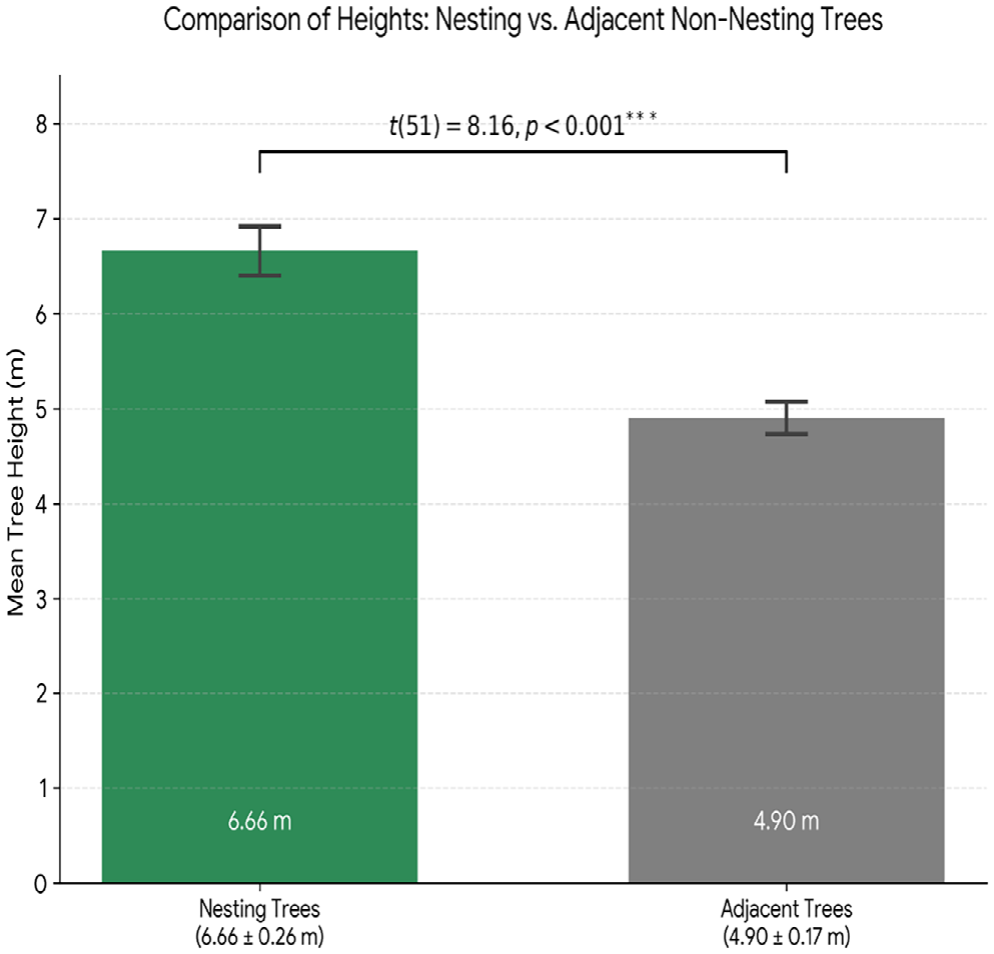
Comparison of tree heights between nesting sites and adjacent non‐nesting trees for *P. mahali* at Chemeron, South Baringo, Kenya (*p* > 0.001). *** They denote the difference in mean tree heights between the two groups is statistically significant at very high level of confidence.

In accordance with our observations, the White‐browed Sparrow‐weavers ( *P. mahali* ) do not randomly choose host trees but rather show a clear tendency to select taller canopy structures. For nesting trees, there was a significant difference between them and adjacent non‐nesting trees in height (*p* < 0.001, Figure 13). This preference for height may be an adaptive trait for predator avoidance since sentinels are able to see terrestrial and aerial predators from a greater distance in taller trees, which is a key aspect of the species' cooperative breeding system (Ferguson and Siegfried 1989). Collias and Collias (1978) also reported that communal nesting birds tend to nest at higher elevations where predation and colony watching are more likely. Similarly, Dean and Milton (1992) showed that sociable and cooperative passerines tend to select taller thorny trees, as they provide the ability to detect predators and enhance nesting security.

Existing literature shows that *P. mahali* is densely populated in dry regions with woodland shrublands and/or wooded grasslands (BirdLife International 2021; Stevenson and Fanshawe 2002; Zimmerman et al. 1999). In this study, it was found that this bird species primarily nests on *Acacia tortilis* and *Acacia elatior*. Despite the presence of other *Acacia* species ( *A. nilotica* , *A. mellifera* , *A. senegal* , *A. brevispica*, *A. hockii*, *A. reficiens*) and other large trees such as *Balanites aegyptiaca* and *Boscia angustifolia*, these two species were specifically selected. They can be attributed to their distinctive physical structure, which is large, tall, and broad with wide canopies that create the “scaffolding” for the communal nests. Also, the large thorns these species have act as a mechanical deterrent and limit the mobility and access of predators that are attracted to the trees. Other studies further attest to the preference for tall trees with larger canopies for bird nesting sites (Jayapal et al. 2009; Kim and Koo 2009). Total basal area was found to be an important factor affecting nesting density, indicating that the removal of mature vegetation would fundamentally impact breeding site stability (Justice and Nyemwerai 2015).

### 4.3 Vertical Nest Placement and Microclimate Regulation

Nesting sites are determined by habitat quality and are an essential element of the study and conservation of birds (Justice and Nyemwerai 2015). Nest site selection is not random but an adaptation influenced by factors such as predation, sunlight, temperature, rainfall, and wind (Galvis et al. 2014). The microhabitat preferences (predation and, to a greater degree, water availability) appeared to affect the placement of nests during this study. All nesting colonies were located within a distance of 350 m of water. This proximity would probably be used as a means to maximize food availability, since food production is generally highest in riparian and near water areas in ASAL environments such as Baringo, thus decreasing foraging trip costs. Reproductive success or survival was not directly observed, but it is hypothesized that reproductive success and survival are promoted by balancing resource availability with protection from threats at these sites (Ferguson and Siegfried 1989). Specifically, the preference for trees well above the general level of ground cover implies an anti‐predatory adaptation; the elevation of nests places the young *P. mahali* further away from ground‐based climbing predators. The height of trees and the actual physical obstacle of the thorns of *A. tortilis* and *A. elatior* probably constitute a major deterrent since explicit predation trials were not carried out (Collias and Collias 1978; Dean and Milton 1992).

**FIGURE 14:**
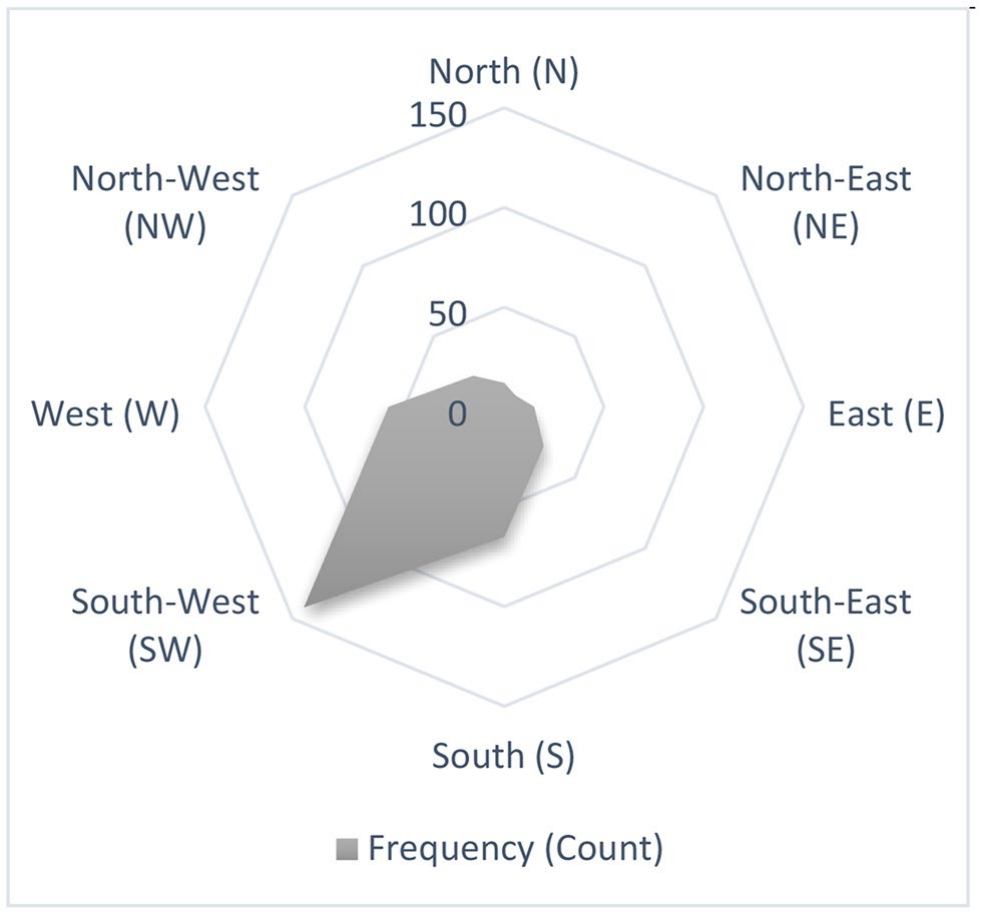
Radar Chart Directional Orientation of *P. mahali* Nests at Chemeron, South Baringo, Kenya; *n* = 350, mean = 232° (South West aspect) (*p* > 0.001).

At the time of this study, nest location was always on the leeward side of the canopy. Nest placement was uniformly leeward of the canopy as quantified in the radar chart (Figure 14), and there is a strong concentration (40.6%) on the South‐West aspect. This is an important strategy for survival as nests are turned away from the prevailing wind and the birds take advantage of the shade provided by the dense foliage of *A. tortilis* and *A. elatior* (Ardia et al. 2006; Murray and Best 2014). This indicates that the “full explanation” of species preference is the result of the interaction between anti‐predator defense (thorns/height), energetic efficiency (food/water proximity), and microclimate regulation (canopy density/sunlight).

This is an interesting study to note that there was a significant difference in nest height between the five transects (*F*_(4,47)_ = 5.17; *p* = 0.002; Figure 6) although there was no significant difference in the height of the host trees (*p* = 0.82). This separation of nest height from tree height strongly suggests that nest height is not a sampling artifact of the availability of trees, but rather a biologically meaningful choice for nest height. In Transects 3 and 5, nests were located at a significantly greater height than in other transects. We suggest that this is an adaptive “vertical avoidance” response. Higher nests may be due to local disturbance or the presence of more ground predators which can prey on *P. mahali* colonies. Also, in open shrublands, higher canopy position may allow for better sight lines and earlier detection of approaching predators. Or, it could be caused by micro‐climatic gradients, with higher nests enjoying more cooling from the wind in hotter, more exposed parts of the study area, and lower nests in other transects benefiting from the thermal buffering effect of thicker mid‐canopy. The adaptation of *P. mahali* within the visually uniform environment is an example of vertical plasticity. This pattern is consistent with previous studies highlighting the role of environmental heterogeneity and behavioral flexibility in nest‐site selection (Mainwaring et al. 2014; Ridley and Raihani 2008).

### 4.4 Colony Composition and Social Stability

This distribution of nest types observed indicates a high level of social stability in the study area. Previous studies have shown breeding nests to have a higher reproductive investment, but this study found that very few nests were used in breeding (11%) and that a large number (89%) were used for roosting purposes (Ferguson and Siegfried 1989). A disproportionately high number of roosting nests means that this area of study under DRTEC, Chemeron is not a transient seasonal breeding ground, but a permanent, year‐round communal home base. The discovery fits with the fact that *P. mahali* lives in rather structured social groups where familial heat regulation and predator defense are of greater importance than reproducing continuously (BirdLife International 2024; Collias and Collias 1978). In addition, the amount of time and effort invested in semi‐permanent roosting structures highlights the importance of habitat stability; these colonies are established social structures and territories, so the elimination of a few favorite host trees can have a significant impact, causing a loss of many years of social hierarchy and territory maintenance. This argument is reinforced by long‐term behavioral and ecological data (BirdLife International 2024; Heinsohn 1992) indicating *P*. *mahali* colonies depend greatly on habitat stability.

### 4.5 Management Implications and Conclusion

This study revealed a clear preference of the White‐browed Sparrow‐weavers in nesting on *Acacia tortilis* and *Acacia elatior* which are currently over‐exploited for charcoal production, wood fuel extraction and building materials. Baringo County Government, along with Kenya Forest Service (KFS) and Kenya Forestry Research Institute (KEFRI) should prioritize protecting those trees that have large DBH values which will serve as sources of seeds and nesting grounds for rejuvenation of the ecosystem. These mature trees are important for the habitat structure that supports communal nesting; therefore, their removal is a loss of function of the habitat for the local avifauna. This specific conservation is crucial for maintaining the integrity of the local ecosystem and protecting biodiversity. Baringo County Government needs to put in place robust livestock and wood fuel management and monitoring programs to reverse the loss of habitats. Ensuring alternative livelihoods and sustainable energy sources such as the use of solar and biogas that do not require the utilization of ecologically important Acacia stands should be a priority for stakeholders and development partners.

The study concludes that habitat features like tree diameter, tree height, water availability and food resources are significant factors that affect the nesting site selection of the *Plocepasser mahali* bird species. The large thorny trees were predominantly *Acacia tortilis* and *Acacia elatior*, and they created suitable habitat for roosting and breeding nests. We suggest that their choice of these species may be a strategic choice to maximize protection from strong winds, direct sunlight and predators, thus potentially increasing the reproductive success of the colony. Distance to water and food resources was the most important habitat factor in determining nest site. Importantly, the results presented here have shown that nesting density of *P. mahali* can be used as a reliable bio‐proxy of rangeland health in semi‐arid ecosystems. Even if this work is confined to Baringo South, the approach and findings may be implemented elsewhere in the Afrotropical belt, where the anthropogenic pressure on woody vegetation is increasing. Local governments can use stability measurement of these bird populations to receive information about the effectiveness of landscape restoration and conservation and create a larger body of evidence.

## Author Contributions

**Enock Nyamorambo Nyamira:** conceptualization (equal), formal analysis (equal), methodology (equal), project administration (equal), resources (supporting), software (equal), supervision (supporting), validation (equal), visualization (equal), writing – original draft (equal), writing – review and editing (equal). **George Morara Ogendi:** conceptualization (equal), formal analysis (lead), investigation (lead), methodology (equal), project administration (equal), resources (lead), software (equal), supervision (lead), validation (equal), visualization (equal), writing – original draft (equal), writing – review and editing (equal). **Rhoda Nyasuguta Ondieki:** conceptualization (equal), formal analysis (equal), investigation (equal), methodology (equal), project administration (equal), software (equal), writing – original draft (equal), writing – review and editing (supporting).

## Funding

The authors have nothing to report.

## Conflicts of Interest

The authors declare no conflicts of interest.

## Supporting information


**Data S1:** Sheet Metadata: Nest Heights Summary.

## Data Availability

All the required data are uploaded as Supporting Information.
